# Establishing the Digital Health Equity & Literacy Program (D-HELP): a student-led initiative to address digital health literacy gaps among emergency department patients at rush

**DOI:** 10.1093/jamiaopen/ooag015

**Published:** 2026-03-06

**Authors:** Qianyi Pu, Ryan Guidi, Tejas C Sekhar, Tina Y Ting, Jules A Tsanang, Aisha Zanib, Lily Noonan, Catherine Chang, Nicholas Cozzi, Eric P Moyer, Galeta Carolyn Clayton

**Affiliations:** Rush Medical College, Rush University Medical Center, Chicago, IL 60612, United States; Rush Medical College, Rush University Medical Center, Chicago, IL 60612, United States; Rush Medical College, Rush University Medical Center, Chicago, IL 60612, United States; Rush Medical College, Rush University Medical Center, Chicago, IL 60612, United States; Rush Medical College, Rush University Medical Center, Chicago, IL 60612, United States; Rush Medical College, Rush University Medical Center, Chicago, IL 60612, United States; Rush Medical College, Rush University Medical Center, Chicago, IL 60612, United States; Rush Medical College, Rush University Medical Center, Chicago, IL 60612, United States; Department of Emergency Medicine, Rush University Medical Center, Chicago, IL 60612, United States; Department of Emergency Medicine, Rush University Medical Center, Chicago, IL 60612, United States; Department of Emergency Medicine, Rush University Medical Center, Chicago, IL 60612, United States

**Keywords:** digital health equity, quality improvement, patient portal utilization, digital literacy, bilingual education, structured educational intervention

## Abstract

**Objective:**

To describe the development and early implementation of the Digital Health Equity & Literacy Program (D-HELP), a student-led quality improvement initiative to promote digital health engagement in the emergency department (ED).

**Materials and Methods:**

Trained student volunteers at Rush University Medical Center delivered in-person education on Epic MyChart and Rush On Demand telehealth services in English and Spanish. Eligible adult patients were identified through the EHR and engaged when clinically appropriate.

**Results:**

Over 4 months, 94 patients were approached, with 64 (68%) patients receiving some level of intervention. Volunteers documented encounter type, interpreter use, and unsolicited patient feedback. MyChart invitations were sent to 27 patients, with 7 registering on-site.

**Conclusion:**

D-HELP demonstrated feasibility, flexibility, and strong patient receptiveness in the ED setting. The model’s low-resource, student-driven design supports scalability and provides a framework for expanding digital health literacy initiatives across diverse clinical settings while addressing social determinants of digital access.

## Background and significance

EHRs have become integral to healthcare delivery, with widespread adoption across the United States. In 2021, 96% of non-federal acute care hospitals and 78% of office-based physicians used certified EHR systems.^1^ Among the most impactful features is the patient-accessible portal, which offers 24/7 access to health information, test results, scheduling, messaging, and telehealth services.^1^^,^^2^ The 21st Century Cures Act in 2016 mandated full, free access to electronic health information to enhance patient autonomy and continuity of care.^3^ COVID-19 further accelerated reliance on digital tools. However, in 2022, only 57% of patients reported accessing their online medical records, and ∼20% lacked portal accounts.^4^

Barriers reflect social determinants of health—including limited literacy, language, income, and minority status—and are worsened by usability challenges.^5–12^ Utilization remains lower among Hispanic, Latinx, and non-Hispanic Black patients.^13^^,^^14^

Recognizing digital literacy as a modifiable social determinant of health, the Digital Health Equity & Literacy Program (D-HELP) was developed as a quality improvement (QI) initiative at Rush University Medical Center’s Emergency Department (ED). D-HELP delivers education on Epic MyChart and Rush On-Demand telehealth services through structured, in-person sessions led by trained student volunteers. Additionally, patients receive brochures and QR-linked written tutorials in English and Spanish.

While prior work has shown that portal training can improve utilization, few efforts have targeted the ED. D-HELP seeks to reduce disparities and improve access by integrating digital literacy support directly into routine ED workflows without adding overhead, disrupting care, or complicating existing workflows.

## Objectives

The paper describes the development, implementation, and early outcomes of D-HELP, a student-led QI initiative established in the Rush ED to address gaps in digital health literacy and patient portal utilization. Specifically, this study aims to:

1. Describe the implementation of a structured, educational intervention in the ED to support patient navigation of Epic MyChart and Rush On-Demand telehealth services.
2. Assess the feasibility, scalability, and reproducibility of delivering digital health literacy support through trained student volunteers.
3. Describe early experience and patient engagement outcomes associated with the intervention
4. Identify opportunities for future implementation and optimization of digital health literacy interventions across diverse clinical settings.

## Implementation of a structured educational intervention

**Figure 1.**
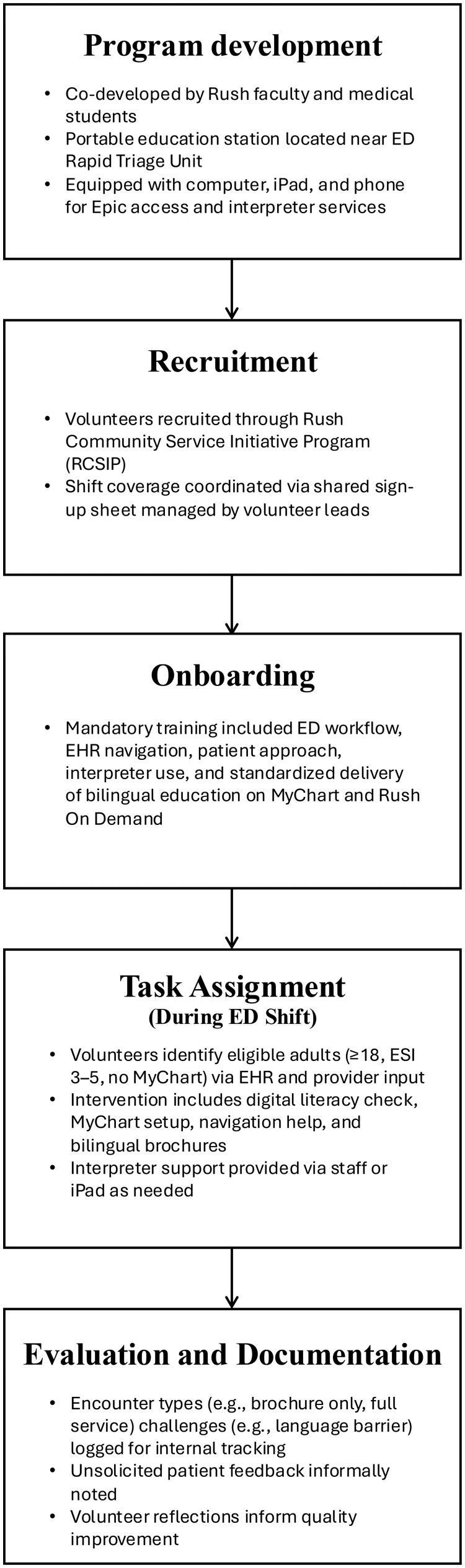
D-HELP Program workflow. Workflow diagram illustrating the Digital Health Equity & Literacy Program (D-HELP) in the emergency department. The figure outlines five sequential stages: (1) Program development, co-led by Rush faculty and medical students using a portable education station near ED rapid triage; (2) Recruitment of volunteers through the Rush Community Student Initiative Program with coordinated shift scheduling; (3) Onboarding, including mandatory training on ED workflow, EHR navigation, interpreter use, and standardized bilingual education; (4) Task assignment during ED shifts, where volunteers identify eligible adult patients without MyChart access and provide digital literacy assessment, account setup, navigation assistance, and interpreter-supported education; and (5) Evaluation and documentation, including logging encounter types, challenges, patient feedback, and volunteer reflections to support quality improvement.

The intervention was designed to be student-led, replicable, and integrated into ED workflows (Figure 1). Together, physicians and students mapped the ED workflow, identified opportunities to incorporate D-HELP without disrupting patient care, and co-developed the educational content. Key components of the program are outlined below:

## Program setting

D-HELP was implemented in the ED at Rush University Medical Center (RUMC). A portable digital health education station was established near the exit of the ED. A portable station equipped with a computer, iPad, and phone provided volunteers with access to Epic and interpreter services. Volunteer shifts occurred from 4-8pm on weekdays.

## Student volunteer recruitment and onboarding

Clinical volunteers, including students from nursing, physician assistant, translational science, and medical programs, were recruited through the Rush Community Service Initiatives Program (RCSIP) at Rush University. All student participants completed a 1.5-hour structured, in-person training led by student leaders alongside ED physicians and residents. Training covered ED workflow, interpreter use, EHR navigation, standardized scripts, and documentation.

To ensure respectful interactions with a diverse population within the ED, the training incorporated cultural competency and professionalism modules. These included strategies for communicating with patients with limited digital or health literacy, use of plain-language and teach-back techniques, motivational interviewing skills, and appropriate utilization of interpreter services. Role-play scenarios allowed volunteers to practice bias-aware communication and patient-centered engagement.

For each shift, at least one student leader was present to provide on-site guidance and ensure consistency in workflow.

## Patient identification

Patient MyChart activation statuses were discernible through Epic EHR. Adult ED patients (18+) with an Emergency Severity Index (ESI) score of 3–5 who were eligible for a patient portal account and did not have an activated MyChart account were considered for participation. The ESI is a 5-level nursing driven ED triage system used to categorize patients based on acuity and anticipated resource needs. Patients with ESI score 3–5 are generally clinically stable, require fewer time-sensitive interventions, and are appropriate for non-clinical educational engagement without disrupting care. Volunteers also utilized real-time judgement when entering the room for an educational encounter. Patients with psychiatric complaints or active MyChart accounts were excluded.

Patients were prioritized based on a combination of clinical stability, anticipated discharge timing, and accessibility for education, as judged by the volunteer after chart review and discussion with the care team as applicable.

## Intervention delivery

Volunteers began each interaction by assessing the patient’s prior experience with digital health portals through a brief series of standardized verbal questions embedded in the patient-approach script. These questions asked about the patient’s familiarity with MyChart, frequency of using MyChart or other electronic platforms, and whether they perceived portal navigation as easy or difficult. While a MyChart sign-up invitation is automatically sent during ED registration at our institution, volunteers offered to resend the invitation based on the patient’s needs and guided patients through account setup. After assisting the patient with setting up or accessing their MyChart account on their preferred device (phone, tablet, or computer), volunteers used their own MyChart accounts on a D-HELP device to model each step in real time. Instructions covered key MyChart features, including scheduling, messaging, test results, billing, and prescription refells. Volunteers spent additional time on features that patients found difficult, while streamlining instructions for those already familiar with basic functions.

**Figure 2.**
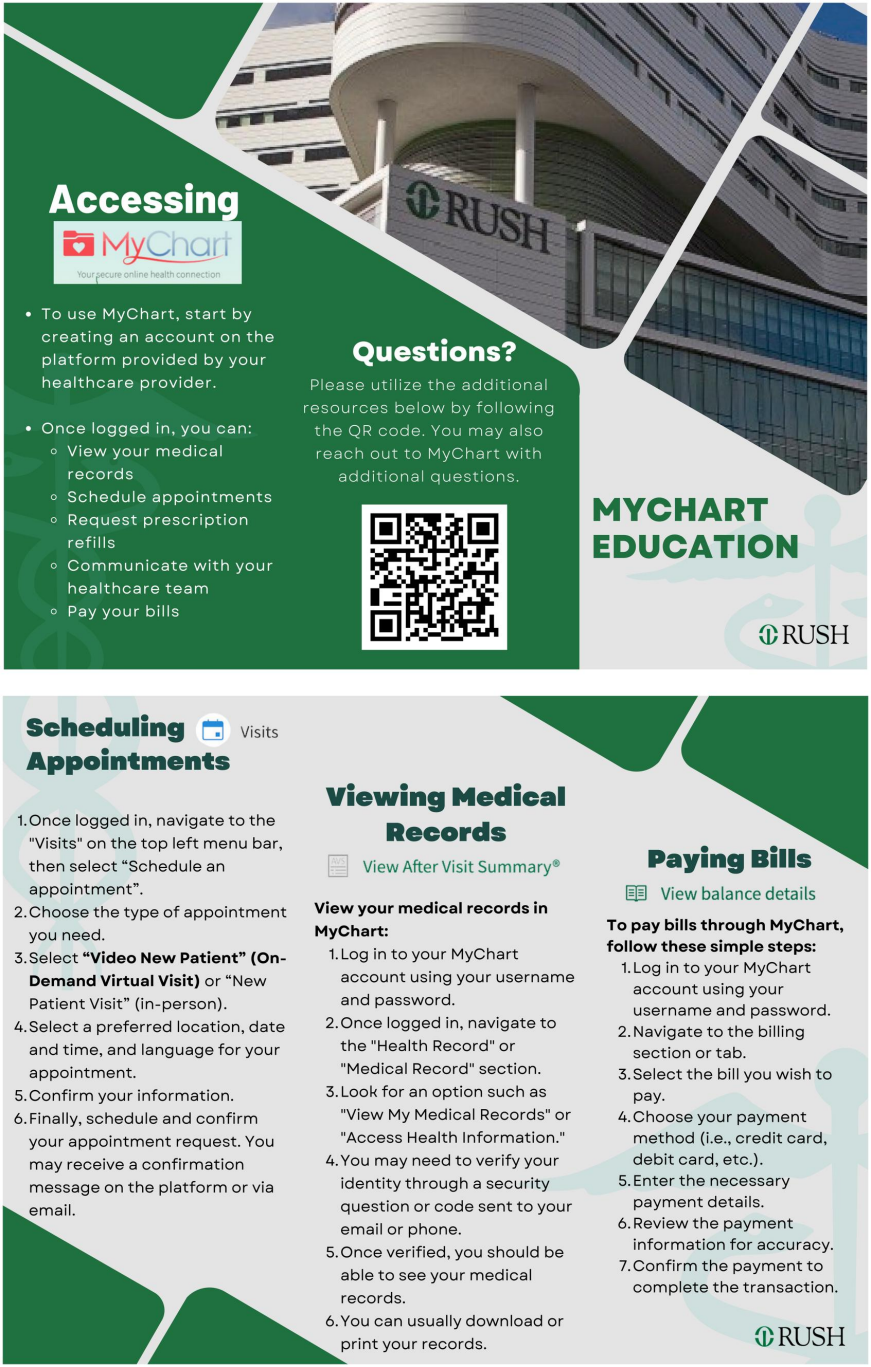
MyChart instruction brochure. Two-page patient-facing MyChart education brochure developed by D-HELP. The brochure explains how to create and use a MyChart account, including viewing medical records, scheduling appointments (in-person or virtual), requesting prescription refills, communicating with the care team, and paying bills. Visual elements include Rush branding, screenshots, step-by-step instructions, and a QR code linking to additional MyChart resources.

At the end of the visit, patients received printed brochures in English or Spanish, summarizing the main MyChart functions (Figure 2). Each brochure included QR codes linking to illustrative, step-by-step written tutorials for key MyChart features. During the intervention, volunteers demonstrated how to scan and use QR codes to ensure accessibility for patients unfamiliar with this feature. While we acknowledge that access to mobile data or Wi-Fi may vary, the inclusion of QR codes provided a scalable solution to deliver in-depth support content without resource overload from patient-centric and financial consideration perspectives. These materials were designed to support continued independent use of MyChart after the encounter, allowing patients to revisit instructions at their own convenience and pace.

To ensure language accessibility, all volunteers had access to both in-person ED interpreter services and iPad-based video interpreter application certified by the institution. The iPad interpreter service provided real-time access to certified medical interpreters in multiple languages.

## Evaluation and documentation

To support quality improvement, volunteers documented each patient interaction digitally during their ED shifts using a standardized data-entry form created for this project. This included the number of patients approached, whether the intervention was delivered, the level of engagement (eg, brochure only vs full support), interpreter use, and any observed barriers or workflow interruptions. All recorded information was de-identified and collected solely for internal QI purposes. Unsolicited patient feedback was informally noted during encounters. These observations were reviewed during periodic team debriefs and informed iterative adjustments to the intervention, including refining patient-approach scripting, modifying the brochure content, and optimizing volunteer workflows to ensure the intervention remained feasible, patient-centered, and compatible with ED operations.

## Feasibility, reproducibility, and scalability of the volunteer-based workflow

D-HELP demonstrated strong feasibility and operational flexibility during its initial phase. Student volunteers integrated smoothly into the ED environment, coordinating with clinical staff and independently identifying eligible patients through EHR review. Over 4 months, 68% (64/94) of the patients approached received at least some form of intervention, reflecting consistent opportunities for engagement during active volunteer shifts. Volunteers routinely adapted the depth and focus of instruction based on patient availability, language needs, and digital fluency, and were trained to approach only well-appearing patients of ESI 3-5 when they were waiting or otherwise unoccupied. Only one encounter was stopped prematurely due to clinical care intervention, underscoring compatibility with existing ED workflows (Table 1). Future iterations may benefit from systematically tracking the duration of volunteer-patient encounters to better assess feasibility and optimize workflow integration.

**Table 1.** Summary of D-HELP implementation metrics and observations.

Category	Metric/observations
Total patients approached	94
Intervention delivered	64/94 (68%)
Brochure only	24/64 (38%)
MyChart invitation only	9/64 (14%)
Full service (eg, brochure, invitation, navigation support)	31/64 (48%)
Encounters interrupted due to clinical care	1/94 (1%)
Encounters with language barriers	3/94 (3%); 1 Cantonese, 2 Spanish
Declined intervention	30/94 (32%)
Common reasons for non-participation (informally noted by volunteers)	Prior familiarity with MyChart, Lack of interest, Preference for non-digital management, Inappropriate clinical/emotional state, Family member managed care, No smartphone access, Clinical care interruption, Unspecified
MyChart invitations sent	27
On-site MyChart registrations	7/27 (26%)
Unsolicited feedback themes	Limited awareness of Rush MyChart and Rush On Demand; High receptiveness to in-person guidance; Appreciation for support received

All observations reflect informal feedback and program delivery data collected as part of routine quality improvement efforts. No formal survey or research instrument was administered.

The standardized structure of the program supported reproducibility across shifts and among volunteers. All participants completed standardized training before their first shift, and each shift is staffed by an experienced student leader. With a steady supply of passionate student volunteers recruited through RCSIP, the program maintained reliable coverage throughout the week. Collectively, these elements allowed the intervention to be delivered consistently.

D-HELP was designed to be both low-resource and scalable, requiring minimal physical infrastructure and use of readily available resources in the ED, including portable computers and iPads. These tools provided real-time access to the EHR and interpreter services as needed. Of the 94 total patient encounters, 3 were reported to involve language barriers (1 Cantonese, 2 Spanish) that were easily able to be accommodated with the use of interpreter services, highlighting the accessibility and feasibility of the intervention under routine ED conditions (Table 1).

## Patient experience and outcomes

During the first 4 months, volunteers engaged 94 adult ED patients. of whom 64 patients (68%), while the remaining 30 (32%) declined, most often citing reasons such as prior familiarity with MyChart, lack of interest, or a preference for non-digital management. Among those who received the intervention, encounter types were documented: 24 patients (38%) received brochures only, 9 (14%) received a MyChart invitation without further support, and 31 (48%) received full services, including brochures, MyChart invitation, and hands-on navigation support.

Informal discussions during the intervention revealed that many patients had limited awareness of digital health tools, were unfamiliar with MyChart, or had only used portals at other institutions.

Volunteers sent 27 MyChart invitations and provided account activation guidance. Seven patients registered on-site, reflecting immediate engagement. Patient receptiveness was consistently positive. Unsolicited patient feedback often included expressed appreciation for the provided support and encouragement to continue the initiative (Table 1). Volunteers and faculty members also noted that brochures were often perceived as too text-dense, and that incorporating more visual elements could improve clarity and usability. Such reflections on barriers and successes were used to inform ongoing quality improvement. No formal analysis was performed.

These findings underscore the value of in-person digital health education and highlight significant gaps in awareness among ED patients. Patient receptiveness suggests a meaningful opportunity to advance digital inclusion through low-barrier, supportive interventions at the point of care.

## Opportunities for future implementation and optimization

D-HELP’s early success highlights opportunities for future growth and refinement, both within and beyond the ED. As the program evolves, future efforts could focus on addressing limitations of the intervention, including language barriers and low rates of engagement. Intervention brochures are currently offered only in English and Spanish, limiting engagement from patients who speak other languages. Additionally, the initial brochure is considered to be text-heavy and may pose challenges for individuals with lower literacy levels. Collaborating with our institution’s Marketing and Communications team to redesign the brochure using plain-language principles and simplified graphics can enhance readability and user-friendliness. Expanding this intervention to include additional languages and adopting a more accessible design will allow us to engage a larger portion of Rush’s diverse patient population. Incorporating light-touch follow-up such as MyChart notifications or text prompts can also support continued engagement. Over time, longitudinal data on MyChart activation and use may offer insights into sustained engagement and inform institutional strategies to advance digital health equity.

Importantly, D-HELP can serve as an infrastructure for assessing digital health inequities, including differences in digital literacy, language needs, device, access, and patterns of portal engagement across demographic groups. Planned next steps include obtaining IRB approval to conduct structured surveys, thereby enabling a more formal assessment of patient population, digital literacy, perceived barriers, opportunities for targeted support, and effectiveness of this intervention. This data will inform future refinements and enhance the ability to deliver patient-centered, equity-focused digital interventions.

Looking ahead, the D-HELP model has strong potential for adaptation across departments, clinics, and affiliated health systems. Its low-resource design, use of common technologies, and trained student volunteers make it well-suited for replication in diverse clinical settings. The standardized workflow and educational materials support easy integration, while offering medical students a meaningful opportunity for service-based learning and digital literacy training.

## Data Availability

The data used in this article will be shared upon reasonable request to the corresponding author.
